# Ethnic Disparities in Use of Bariatric Surgery in the USA: the Experience of Native Americans

**DOI:** 10.1007/s11695-020-04529-w

**Published:** 2020-03-18

**Authors:** Ibrahim Al-Sumaih, Nga Nguyen, Michael Donnelly, Brian Johnston, Zhamak Khorgami, Ciaran O’Neill

**Affiliations:** 1 Centre for Public Health, School of Medicine, Dentistry and Biomedical Sciences, Institute of Clinical Sciences, Queen’s University Belfast, Block B, Royal Victoria Hospital, Belfast, BT12 6BA UK; 2 Ministry of Health, Riyadh, Saudi Arabia; 3 Belfast Health and Social Care Trust, Belfast, UK; 4 Department of Surgery, College of Medicine, University of Oklahoma, Tulsa, OK USA; 5 Harold Hamm Diabetes Center, The University of Oklahoma Health Sciences Center, Oklahoma City, OK USA

**Keywords:** Bariatric surgery, Obesity, Ethnic groups, Health expenditure, Insurance, Length of stay, American Indians

## Abstract

**Purpose:**

To examine disparities in use of bariatric surgery in the USA with particular focus on the experience of Native Americans.

**Materials and Methods:**

Multivariable logistic regression models were applied to the hospital discharge HCUP-NIS dataset (2008–2016) in order to examine the influence of ethnicity in use of bariatric surgery while controlling for aspects of need, predisposing and enabling factors. Separate models investigated disparities in length of stay, cost and discharge to healthcare facility among patient episodes for bariatric surgery.

**Results:**

Full data for 1,729,245 bariatric surgery eligible participants were extracted from HCUP-NIS. The odds of Native Americans receiving bariatric surgery compared to White Americans were 0.67 (95% CI, 0.62–0.73) in a model unadjusted for covariates; 0.65 (95% CI, 0.59–0.71) in a model adjusted for demography and insurance; 0.59 (95% CI, 0.54–0.64) in a model adjusted for clinical variables; and 0.72 (95% CI, 0.66–0.79) in a model adjusted for demographic, insurance types and clinical variables. Native Americans who underwent surgery had significantly shorter lengths of stay, lower healthcare expenditures and lower likelihood of discharge to other healthcare facilities relative to White Americans (controlling for covariates).

**Conclusion:**

Our study, the first study to examine this subject, showed apparent variations in receipt of bariatric surgery between Native Americans and White Americans even after a range of covariates were controlled. In addition, Native Americans have shorter lengths of stay and significantly lower expenditures.

**Electronic supplementary material:**

The online version of this article (10.1007/s11695-020-04529-w) contains supplementary material, which is available to authorized users.

## Introduction

There is a significant economic burden associated with obesity that includes the cost of managing the condition and associated co-morbidities [1], its impact on health-related quality of life HRQoL [2] and its impact in terms of lost productivity [3]. These costs have risen as the prevalence of obesity has increased. In the USA, between 1988–1994 and 2015–2016, for example, the prevalence of obesity among adults rose from 22.9 to 39.6% (State of Obesity, 2019). In 2000–2005, the healthcare costs associated with obesity were estimated to be $210 billion per year, approximately 21% of US healthcare expenditures [4], and it was estimated that medical costs will increase by $44–66 billion per year by 2030 [5]. The experience in other developed countries while perhaps not as stark has nevertheless been similar [6]. Public health efforts to stem the rise in obesity have met with limited success [7, 8] though interventions that are targeted at the individual level have shown greater promise. Bariatric surgery appears to be a safe and cost-effective means of achieving weight loss [9]. In the USA, the uptake of bariatric surgery increased from 2.7 per 100,000 procedures in 1990 to 54.2 per 100,000 in 2008 among adults aged 18 and over [10] before plateauing through to 2012 at around 51 per 100,000 [11].

Previous work has looked at variations in expenditure by type of procedure [12], in uptake between men and women [10], between different ages and insurance status categories [13] as well as across distinct geographic areas with different levels of population need [14, 15]. This work has shown, inter alia, marked changes over time in the type of procedure that has been used in the USA (laparoscopic surgery increasing from 2.1 of procedures in 1998 to over 90% of procedures in 2008) [10]; women were consistently more likely than men to have surgery constituting around 80% from 1998 to 2012 [10, 11]; Whites were more likely to receive surgery than Blacks [16]; population need – in terms of prevalence of obesity at the state level – had little relationship with the uptake of surgery [14, 15]; there has been a reduction in complications associated with surgery over time [17]; and variations in surgery costs were related to co-morbidity, complexity and length of hospital stay [12].

Native American adults are 50% more likely to be obese than non-Hispanic Whites, and they have higher age-adjusted odds for diabetes, heart disease and stroke of 2.4, 1.3 and 1.8 than Whites, respectively [18]. Despite these facts, no published study has examined the use of bariatric surgery by Native Americans relative to other ethnic groups. This may in part be explained by their relatively small size as a proportion of the population. While the percentage of Native Americans who are obese is similar to Blacks [19], Blacks constituted 13.4% of the population in 2018, Hispanics or Latino 18.3%, Asians 5.9% and Americans Indians and Alaska Natives just 1.3% of the population [19]. Inevitably, the small number of Native Americans in surveys makes it more difficult to investigate comparative ethnic group experiences. Nevertheless, the lack of research with respect to this group gives rise to a clear gap in our understanding regarding their comparative experience.

In this paper, we report the results of an examination of the uptake of bariatric surgery between 2008 and 2016 among obese adults using the nationally representative inpatient data HCUP-NIS. We give particular emphasis to the comparative experience of Native Americans. We compare uptake between different ethnic groups controlling for a range of covariates including co-morbidity, gender, age, location and insurance status and time using data pooled on over two million episodes of inpatient care.

## Methods

### Data Source

Data were obtained from the Healthcare Cost and Utilization Project-National (Nationwide) Inpatient Sample (HCUP-NIS) and Agency for Healthcare Research and Quality (AHRQ). HCUP-NIS provides information on all patients, regardless of payer from a 20% stratified sample of all discharges from US hospitals, excluding rehabilitation and long-term acute care hospitals [20].

### Participants

The International Classification of Diseases, 9th Revision and 10th Revision (ICD9 and ICD10) primary diagnostic and procedure codes were used to identify all admissions eligible for bariatric surgeries. In the main analyses, these comprised episodes of care with a diagnosis of morbid obesity (ICD9 278.01, ICD10 E66.01) with type II diabetes or hypertension (for details on ICD codes use, please visit Appendix 1) [21]. Although HCUP-NIS provides data on primary and a range of secondary diagnoses or procedures, primary diagnosis is the condition chiefly responsible for hospitalization and was used to identify the cohort used in this study. ICD9 codes were used for HCUP-NIS data before quarter 4 of 2015, and ICD10 codes were applied to data afterward. We pooled 9 years data from 2008 to 2016 to increase sample size and allow analyses across different subgroups.

From the eligible cohort of morbidly obese admissions, bariatric surgeries were identified using ICD procedure codes. Bariatric surgeries comprised laparoscopic sleeve gastrectomy (ICD9 43.82, ICD10 0DB64Z3), laparoscopic gastric bypass (ICD9 44.38, ICD10 0D164ZA), open gastric bypass (ICD9 44.39, ICD10 0D160ZA), laparoscopic adjustable gastric band (ICD9 44.95, ICD10 0DV64CZ) and open adjustable gastric band (ICD9 44.69, ICD10 0DV60CZ). Biliopancreatic diversion was not included due to the low number and lack of specific procedure code. The analysis was confined to elective admissions to decrease the likelihood of including surgical corrections or other conditions. Elective admissions with a primary procedure of bariatric surgery identified by these ICD codes and a Diagnosis-Related Group code of procedures for obesity (619, 620 or 621) were defined as those in receipt of bariatric surgery.

An age-adjusted Deyo-Charlson co-morbidity index (ACCI) was generated based on age at admission and diagnostic/procedure codes of each admission to identify whether it included each of the following co-morbidities: congestive heart failure; chronic pulmonary disease; cerebrovascular disease; diabetes mellitus with or without chronic complications; dementia; myocardial infarctions; rheumatic disease; peripheral vascular disease; mild, moderate or severe liver disease; peptic ulcer disease; renal disease; hemiplegia or paraplegia; and HIV/AIDS. The presence of each co-morbid status was then weighted using the Deyo-Charlson algorithm and summed up to give a unique ACCI score [22, 23].

Race/ethnicity was grouped into six categories: White, Black, Hispanic, Asian, Native American and other races/ethnicities based on self-reported status. Sample characteristics were described by ethnicity using proportions for categorical variables, median and interquartile range (IQR) for skewed continuous variables and mean and standard deviation (SD) for other continuous variables.

### Statistical Analysis

Logistic regression models were used to explore the use of bariatric surgeries over time. While focus was given to the ethnicity of inpatients in the models, a range of covariates that might have affected outcomes were used to adjust models. Selection of covariates was informed by the Behavioural Model (BM) of Health Services Use, developed by Andersen [24]. Variables were selected based on the hypothesis that they could predispose, enable or influence the need of the inpatient for surgery, consistent with the three components of BM models – predisposing, enabling and need factors. Predisposing factors included age and gender; enabling factors included insurance type, median household income for patient’s ZIP Code, location – broadly in terms of rurality – and hospital characteristics including hospital’s bed size, ownership/control status and location and teaching status; and need factors were co-morbidity score, type II diabetes and hypertension. In order to generate national estimates using HCUP-NIS data that span multiple years, all models were weighted by trend weight for data years prior to 2012 and by the discharge-level weight for data years 2012 and later. This is consistent with the recommendations of the AHRQ [25].

HCUP-NIS classified the expected primary payer insurance status as Medicare, Medicaid, private insurance and others. Area level income provided a quartile classification of the estimated median household income of residents in the patient’s ZIP Code, ranked from the lowest to highest income populations. Patient location was a six-category urban-rural classification scheme for US counties developed by the National Center for Health Statistics (NCHS) that included “Central” counties of metro areas of > = 1 million population, “Fringe” counties of metropolitan (metro) areas of > = 1 million population, counties in metro areas of 250,000–999,999 population, counties in metro areas of 50,000–249,999 population, Micropolitan counties, Not metropolitan or Micropolitan counties. In order to control for hospital characteristics, the unique HCUP hospital number exclusive to the NIS was used to link the core data to the hospital weights file. These variables comprised hospital bed size (small, medium, large), hospital’s ownership/control category (government/private) and location (urban/rural) and teaching status of hospital (non-teaching/teaching).

Among admissions receiving bariatric surgery, we further explored the potential racial disparities in different hospital outcomes including discharge destination, length of stay in hospital and hospital-incurred costs. We used weighted multivariable logistic regression to explore the likelihood of being discharged to another healthcare facility (including long-term care facilities or care homes, short-term hospitals, home healthcare and other rehabilitation centres) compared to routine discharge (i.e. discharge to home, self-care and court/law enforcement), excluding those who died in the hospital. Models were adjusted for socio-demographic variables (insurance type, ethnicity, gender, age at admission, median household income, location) and clinical variables (age-adjusted co-morbidity score ACCI, hospital teaching status, hospital location, hospital ownership, hospital bed size and number of procedures performed) and bariatric surgery types (laparoscopic sleeve gastrectomy, laparoscopic gastric bypass or others including gastric bands and open gastric bypass surgery). Year was added as a predictor to control for variability over time in all regression models.

Similarly, based on the nature of count data and evidence of overdispersion of length of stay in our data, we fitted weighted negative binomial regression models to examine factors associated with length of stay. Generalized linear models (GLM) were used to accommodate the continuous, positive and skewed nature of hospital cost data in the cost analysis. Akaike information criterion (AIC) and Bayesian information criterion (BIC) were used to assess the fit of the GLM model. The link function and distribution family were jointly chosen using AIC and BIC while running a series of GLM models. Marginal effect analyses were used to estimate the hospital cost. The “cost-to-charge” ratio tool provided by AHRQ-HCUP was used to convert discharges to hospital costs [20]. Hospital costs were then adjusted for inflation using the personal consumption expenditure health component price index based on its ability to capture information on expenditures by all payers [26, 27].

In sensitivity analyses, those with a BMI over 40 without hypertension or type 2 diabetes were added to the cohort considered eligible for surgery. These results are reported in Appendix 2.

Data are available from the authors upon request and with permission of AHRQ. All analyses and data manipulations were performed using STATA software, version 15 (College Station, TX: StataCorp LLC).

## Results

Table 1 presents descriptive statistics of the pooled 9-year cohort used in subsequent analyses. This eligible cohort comprised a total of 1,729,245 inpatient episodes. The percentage of White, Native American, Black, Hispanic, Asian and other ethnicities were 68.8%, 0.7%, 19.3%, 8.4%, 0.7% and 2.1%, respectively.

**Table 1:** Descriptive statistics of the pooled sample

	White American (*n* = 1,188,771)		Native American (*n* = 11,886)		Black American (*n* = 333,424)		Hispanic American (*n* = 145,963)		Asian American (*n* = 12,390)		Other ethnicities (*n* = 36,811)	
	*N*	%	*N*	%	*N*	%	*N*	%	*N*	%	*N*	%
Female	714,362	60.1%	7409	62.3%	244,335	73.3%	94,453	64.7%	7110	57.4%	23,532	63.9%
Age, mean (SD)	59.4	12.9	53.5	13.7	53.7	13.8	54.4	14.8	54.8	14.7	56.0	14.2
Insurance												
Medicare	608,198	51.2%	4769	40.1%	143,579	43.1%	56,995	39.1%	4567	36.9%	14,816	40.3%
Medicaid	132,511	11.2%	2610	22.0%	76,305	22.9%	35,137	24.1%	2721	22.0%	7118	19.3%
Private insurance	372,138	31.3%	2985	25.1%	85,757	25.7%	39,321	26.9%	4274	34.5%	11,744	31.9%
Others	75,924	6.4%	1522	12.8%	27,783	8.3%	14,510	9.9%	828	6.7%	3133	8.5%
Location												
Central counties	240,633	20.2%	2174	18.3%	148,973	44.7%	73,549	50.4%	4957	40.0%	16,722	45.4%
Large metro	303,368	25.5%	1362	11.5%	74,555	22.4%	21,997	15.1%	2146	17.3%	8315	22.6%
Medium metro	248,259	20.9%	2409	20.3%	53,431	16.0%	31,522	21.6%	3474	28.0%	4903	13.3%
Small metro	136,467	11.5%	1385	11.7%	24,920	7.5%	8802	6.0%	581	4.7%	2292	6.2%
Micropolitan	157,299	13.2%	2556	21.5%	19,169	5.8%	7094	4.9%	1043	8.4%	2630	7.1%
Not metropolitan or Micropolitan	102,745	8.6%	2000	16.8%	12,376	3.7%	2999	2.1%	189	1.5%	1949	5.3%
Income												
First quartile	330,690	27.8%	5978	50.3%	174,364	52.3%	60,923	41.7%	2078	16.8%	12,102	32.9%
Second quartile	347,352	29.2%	3058	25.7%	73,591	22.1%	35,286	24.2%	2487	20.1%	8869	24.1%
Third quartile	299,304	25.2%	2007	16.9%	53,321	16.0%	32,657	22.4%	3826	30.9%	9159	24.9%
Fourth quartile	211,425	17.8%	843	7.1%	32,148	9.6%	17,097	11.7%	3999	32.3%	6681	18.2%
Type II Diabetes	768,780	64.7%	8638	72.7%	212,438	63.7%	102,038	69.9%	8798	71.0%	23,931	65.0%
Hypertension	843,061	70.9%	7846	66.0%	233,224	70.0%	97,971	67.1%	7745	62.5%	26,467	71.9%
Hospital characteristics												
Private hospital	1,074,291	90.4%	10,504	88.4%	293,190	87.9%	129,109	88.5%	11,071	89.4%	33,132	90.0%
Hospital in urban area	1043,329	87.8%	9265	78.0%	314,278	94.3%	141,167	96.7%	11,545	93.2%	34,456	93.6%
Teaching hospital	571,814	48.1%	5549	46.7%	212,388	63.7%	79,586	54.5%	6830	55.1%	20,005	54.4%
Hospital bed size												
Small	188,445	15.9%	2018	17.0%	45,458	13.6%	21,822	15.0%	2221	17.9%	5756	15.6%
Medium	328,200	27.6%	3078	25.9%	94,294	28.3%	40,610	27.8%	3361	27.1%	9714	26.4%
Large	672,126	56.5%	6790	57.1%	193,672	58.1%	83,531	57.2%	6808	55.0%	21,341	58.0%
ACCI, median (IQR)	3	(2–6)	3	(2–5)	3	(1–5)	3	(1–5)	3	(2–6)	3	(1–5)
Number of procedures, median (IQR)	1	(0–3)	1	(0–3)	1	(0–2)	1	(0–2)	1	(0–3)	1	(0–3)

Note: Income quartiles presented in this table are the estimated median household income of residents in the patient’s ZIP Code. The quartiles are identified by values of 1 to 4, indicating the poorest (first quartile) to wealthiest populations (fourth quartile)

As can be seen from the table compared to other ethnicities, a higher percentage of inpatients of Native American ethnicity had type II diabetes, lived in rural areas (not metropolitan and micropolitan areas) and were treated in rural areas. Compared with Whites, Native Americans were younger, less likely to have private insurance, more likely to live in poor areas (first, i.e. lowest income quartile) and less likely to be treated in a teaching hospital.

**Fig. 1:**
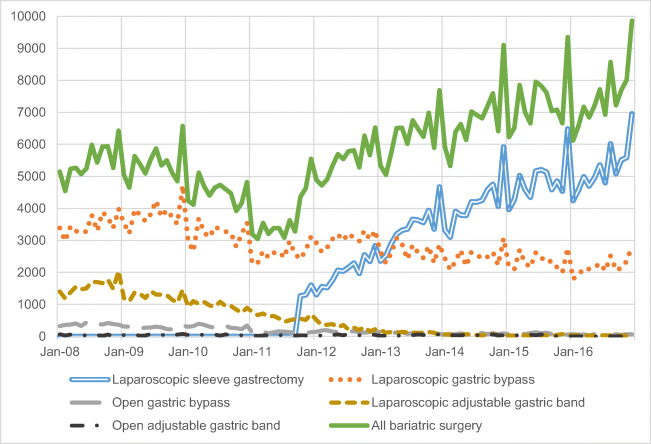
Total number of bariatric surgeries and number of different types of bariatric surgeries over time

In Fig. 1, the number of elective admissions for different types of procedure is shown over time. The figure clearly shows the increasing popularity of laparoscopic sleeve gastrectomy (LSG) (for a variety of reasons including evidence of efficacy, safety and expansion of insurance coverage [12]) over other types of surgery, this one type contributing to much of the overall growth in bariatric surgery since late 2011.

**Fig. 2:**
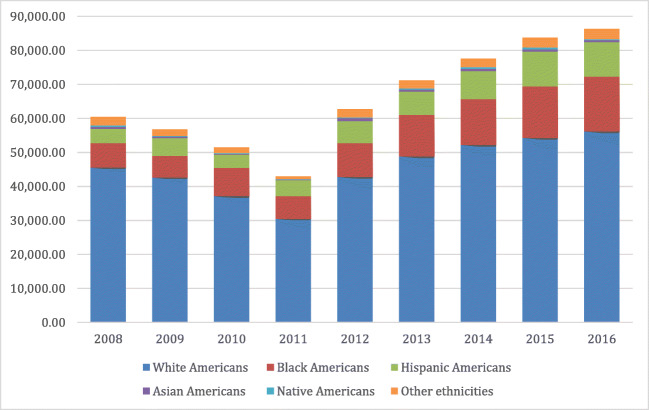
Total number of bariatric surgeries by races/ethnicities overtime

In Fig. 2, the breakdown of surgery by the ethnic group over time is presented, the resurgence in surgeries after 2011 and the increasing proportion of surgeries related to Blacks and Hispanics perhaps being the most notable aspects of the figure. In Fig. 3a–f (appendix 3), trends in specific types of procedure by ethnic group are shown. In brief, these show the increased use of LSG after 2011 and the decline of other types of surgery among this group. Figure 3a shows that Native Americans were slightly slower in the uptake of LSG relative to other ethnic groups.

In Table 2, the likelihood of bariatric surgery among eligible inpatients is reported in a series of models controlling for different combinations of covariates. In each model, the adjusted odds ratio of surgery is significantly lower among Native Americans than Whites. In Model 3, adjusted only for clinical variables, the adjusted odds ratio of surgery among Native Americans is lowest among that of any ethnic group. In Table 3, adjusted odds ratios for a range of outcomes among those in receipt of surgery are reported by ethnic group. As can be seen, Native Americans in receipt of surgery had lower length of stay and lower costs and were less likely to be discharged to another healthcare facility.

**Table 2:** The likelihood of receiving bariatric surgeries among eligible admissions across different races/ethnicities

	Model 1			Model 2			Model 3			Model 4		
	OR	95% CI		OR	95% CI		OR	95% CI		OR	95% CI	
		Lower	Upper		Lower	Upper		Lower	Upper		Lower	Upper
White Americans	Ref			Ref			Ref			Ref		
Native Americans	0.671^***^	0.618	0.728	0.648^***^	0.595	0.707	0.585^***^	0.537	0.637	0.722^***^	0.661	0.79
Black Americans	0.818^***^	0.805	0.831	0.690^***^	0.677	0.702	0.601^***^	0.59	0.611	0.711^***^	0.699	0.725
Asian Americans	1.154^***^	1.081	1.231	0.720^***^	0.67	0.773	0.963	0.899	1.031	0.838^***^	0.779	0.902
Hispanic Americans	1.199^***^	1.175	1.223	1.061^***^	1.038	1.085	0.871^***^	0.853	0.889	1.077^***^	1.053	1.102
Other races/ethnicities	1.597^***^	1.545	1.651	1.357^***^	1.307	1.409	1.280^***^	1.235	1.327	1.365^***^	1.314	1.418

Note: This table presents the results from four sets of logistic regression analysis combined with linear splines without weighting variable. Model 1: unadjusted model. Model 2: adjusted for demographic and socio-economic variables insurance type, gender, age at admission, median household income for patient’s ZIP Code, location. Model 3: adjusted for clinical variables. Model 4: fully adjusted model for all demographic, socio-economic and clinical variables. * *p* < 0.05, ** *p* < 0.01, *** *p* < 0.001. CI, confidence intervals

**Table 3:** Other hospital outcomes among different ethnicities

	Discharge to healthcare facility OR (95% CI)		Length of stay Days (95% CI)		Hospital costs 2016 USD (95% CI)	
	Unadjusted models	Adjusted models ^Φ^	Unadjusted models	Adjusted models ^Φ^	Unadjusted models	Adjusted models ^Φ^
White Americans	Ref	Ref	Ref	Ref	Ref	Ref
Native Americans	0.23* (0.1–0.56)	0.26* (0.11–0.64)	− 0.28* (− 0.36 to − 0.2)	− 0.33* (− 0.41 to − 0.25)	− 595* (− 1050 to − 139)	− 775*** (− 1204 to − 346)
Black Americans	1.49* (1.38–1.61)	1.2* (1.1–1.3)	0.12* (0.1 to 0.15)	0.09* (0.06 to 0.12)	− 33 (− 143 to 77)	378* (277 to 479)
Asian Americans	0.32* (0.18–0.58)	0.5* (0.28–0.9)	− 0.02 (− 0.33 to 0.29)	0.12 (− 0.23 to 0.48)	1873* (995 to 2750)	674 (− 65 to 1412)
Hispanic Americans	0.87* (0.78–0.98)	0.74* (0.65–0.83)	− 0.06* (− 0.09 to − 0.03)	− 0.05* (− 0.08 to − 0.02)	− 441* (− 571 to − 311)	− 41 (− 162 to 80)
Other ethnicities	0.86 (0.72–1.04)	0.79* (0.65–0.96)	0.02 (− 0.03 to 0.06)	− 0.02 (− 0.07 to 0.03)	− 174 (− 430 to 82)	− 11 (− 220 to 197)

Notes: ^Φ^ Models were adjusted for bariatric surgery types (laparoscopic sleeve gastrectomy, laparoscopic gastric bypass and others), insurance type, ethnicity, gender, age at admission, median household income, location, co-morbidity score ACCI, hospital teaching status, hospital location, hospital ownership, hospital bed size and number of procedures performed. * denotes results statistically significant at *p*≤0.05

In Table 4 (appendix 4), the full results of the logistic regression on uptake of surgery (Model 4) are reported.

Full results are reported in Table 4 in the supplement.

## Discussion

Bariatric surgery is a safe and cost-effective treatment of morbid obesity [9]. Its use in the USA has increased steadily since the extension of coverage by Medicare in 2011 as perioperative safety has improved and insurance coverage expanded [10, 11]. Previous studies demonstrated distinct patterns of utilization. The Andersen model (of predisposing, enabling and need factors) may be used to aid our interpretation of these patterns. The higher uptake among females that was found in other studies [13, 14] and echoed here, for example, may relate to gender differences in predisposing factors that are associated with reproduction [28] or social norms around the perception of obesity that tend to be particularly acute for females [29]. Similarly, the differences in uptake between eligible Blacks and Whites that were observed in this study and that have been related previously to insurance status [16] may be interpreted as an enabling factor, while ACCI or the presence of specific conditions may be interpretable in terms of need.

No previous study in this field has examined the comparative experience of Native Americans and potential explanatory factors in this. While Native Americans are a relatively small ethnic group in the USA, this lack of research is surprising given that Native Americans have much higher levels of obesity and obesity-related morbidity than Whites. Our study has shown that, controlling for a range of need, enabling and predisposing factors, eligible Native Americans are approximately 30% less likely to receive bariatric surgery than their White counterparts, a disparity comparable to that of Blacks. In models that controlled solely for clinical factors, the disparity experienced by Native Americans was even sharper (over 40% less likely) and greater than that experienced by Blacks. (Sensitivity analyses that included patients with BMI > 40 without co-morbid hypertension or type 2 diabetes in the eligibility criteria had no material effect on results). Compared with other ethnicities, the slower transition to laparoscopic sleeve surgery among Native Americans (now the dominant form of surgery performed) may provide further evidence of disadvantage in terms of equitable access. Our analyses show that the length of stay among Native Americans and healthcare costs are significantly shorter and lower compared to Whites; the average surgery-related episode cost was $775 less among Native Americans compared to $378 more among Blacks. These findings regarding lower hospital costs may be explained at least partly in terms of Native Americans having a shorter length of stay (approximately 1/3 of a day, Table 3) and being more likely to receive surgery outside urban centres (16.8% compared to 8.6%, Table 1). Our control for ACCI and insurance status suggests, for example, that this difference is unlikely to be related to need or insurance coverage. The fact that Native Americans (for a variety of reasons including historical discrimination) tend to be geographically concentrated and isolated [30] may explain lower uptake. Perhaps, Native Americans find it more difficult to access hospitals that provide bariatric surgery, and they may have been disproportionality affected by the concentration of services in Centers of Excellence following the decision to provide national coverage via the Centers for Medicare and Medicaid in 2006. Why Native Americans exhibit shorter lengths of stay and a lower probability of being discharged to another healthcare facility might also relate to geographic isolation though this is purely speculative. The findings are though consistent with a broader experience of disadvantage among this group in terms of socio-economic status [31], health [32] and access to healthcare [31, 33–37]. The fact that this is the first study to examine the experience of this group in relation to bariatric surgery given their relative need lends further weight to the suggestion that they are an underserved group.

## Limitations

There are a number of limitations to our study. First, we are unable to link inpatient episodes over time to specific individuals or explore issues such a readmissions or changes in BMI following bariatric surgery. Second, we are similarly constrained by the data included in the survey in terms of the ability to control for potentially useful covariates. For example, while we can control for diabetes status, we cannot control for how successfully blood glucose is controlled or how long since diabetes was diagnosed. Similarly, while we can control for age and sex, we have no direct insight into the intentions of women regarding pregnancy. We can use proxy indicators related to place of residence for some covariates such as income – no such proxy exists with respect to indicators of need. Third, we observe only those surgeries performed in an inpatient setting and can make no claims in respect of non-admitted patients. These, however, are limitations imposed on us by the survey and limit the scope of our investigation.

## Strengths

These limitations must be balanced against the great strength of this study which is its sample size. Such a large sample size HCUP-NIS affords that allows us to explore the role of ethnicity for groups whose numbers would preclude analysis in other surveys. Because of this, it has been possible to demonstrate a previously unidentified disparity.

## Conclusions

Our study highlights the existence of disparities in utilization of bariatric surgery among eligible Native Americans relative to Whites in the USA. The disparity is similar in magnitude to that experienced by Blacks and is evident after we control for a variety of potentially contributing factors that include insurance status. The disparity may in part relate to the geographic isolation experienced by this ethnic group. It reflects a broader experience of disadvantage experienced by Native Americans in terms of socio-economic status, health and access to healthcare. It is hoped that this study will both serve to draw attention to a previously unexamined dimension of this experience and help encourage further studies into their use of and access to bariatric services.

## Electronic Supplementary Material

ESM 1 (DOCX 85 kb)
