# Supplementary material for: Ethnic Disparities in Use of Bariatric Surgery in the USA: the Experience of Native Americans
Source: Obes Surg. 2020 Mar 18;30(7):2612–9. doi: 10.1007/s11695-020-04529-w (PMC7260278; doi:10.1007/s11695-020-04529-w)
Supplement: Supplementary file 1 — (DOCX 85 kb) [file 11695_2020_4529_MOESM1_ESM.docx]

**Appendix 1.** ICD 9 and ICD 10 used to identify patients with diabetes type 2 and blood hypertension.

| Obesity-related comorbidity | ICD9 | ICD10 |
| --- | --- | --- |
| Diabetes type 2 | 25000  25002  25010  25020  25040  25050  36641  25060  3572  3559  25070  25080  25030  25090  24920  36202  2721 | E1100  E1101  E1121  E1122  E1129  E11311  E11319  E113211  E113212  E113213  E113219  E113291  E113292  E113293  E113299  E113311  E113312  E113313  E113319  E113391  E113392  E113393  E113399  E113411  E113412  E113413  E113419  E113491  E113492  E113493  E113499  E113511  E113512  E113513  E113519  E113521  E113522  E113523  E113529  E113531  E113532  E113533  E113539  E113541  E113542  E113543  E113549  E113551  E113552  E113553  E113559  E113591  E113592  E113593  E113599  E1136  E1137X1  E1137X2  E1137X3  E1139  E1140  E1141  E1142  E1143  E1144  E1149  E1151  E1152  E1159  E11610  E11618  E11620  E11621  E11622  E11628  E11630  E11638  E11641  E11649  E11665  E11669  E118  E119  E1300  E1301  E1310  E1311  E1321  E1322  E1329  E13311  E13319  E133211  E133212  E133213  E133219  E133291  E133292  E133293  E133299  E133311  E133312  E133313  E133319  E133391  E133392  E133393  E133399  E133411  E133412  E133413  E133419  E133491  E133492  E133493  E133499  E133511  E133512  E133513  E133519  E133521  E133522  E133523  E133529  E133531  E133532  E133533  E133539  E133541  E133542  E133543  E133549  E133551  E133552  E133553  E133559  E133591  E133592  E133593  E133599  E1336  E1337X1  E1337X2  E1337X3  E1337X9  E1339  E1340  E1341  E1342  E1343  E1344  E1349  E1351  E1352  E1359  E13610  E13618  E13620  E13621  E13622  E13628  E13630  E13638  E13641  E13649  E1365  E1369  E138  E139  E781 |
| Hypertension | 4010  4011  4019 | I10 |

**Appendix 2**

The population used in the sensitivity analysis include:

- The main population defined in our Method section, N = 1,729,245
- The SA cohort: those with a diagnosis code of morbid obesity + BMI>40 + (No hypertension/diabetes type 2): N = 367676

In the sensitivity analysis, the logistic regression model for pooled data comprised 2,065,848 observations. R^2^ of the fully adjusted model (Model 4) was 20.16%.

|  | **Model 1** | | | **Model 2** | | | **Model 3** | | | **Model 4** | | |
| --- | --- | --- | --- | --- | --- | --- | --- | --- | --- | --- | --- | --- |
|  | **OR** | **95% CI** | | **OR** | **95% CI** | | **OR** | **95% CI** | | **OR** | **95% CI** | |
|  |  | **Lower** | **Upper** |  | **Lower** | **Upper** |  | **Lower** | **Upper** |  | **Lower** | **Upper** |
| White Americans | Ref |  | | Ref |  | | Ref |  | | Ref |  | |
| Native Americans | 0.627^***^ | 0.584 | 0.675 | 0.635^***^ | 0.588 | 0.685 | 0.539^***^ | 0.499 | 0.581 | 0.692^***^ | 0.64 | 0.748 |
| Black Americans | 0.805^***^ | 0.794 | 0.817 | 0.716^***^ | 0.705 | 0.727 | 0.602^***^ | 0.593 | 0.611 | 0.738^***^ | 0.727 | 0.75 |
| Asian Americans | 1.058 | 0.999 | 1.121 | 0.654^***^ | 0.614 | 0.697 | 0.814^***^ | 0.766 | 0.865 | 0.722^***^ | 0.677 | 0.771 |
| Hispanic Americans | 1.283^***^ | 1.262 | 1.304 | 1.114^***^ | 1.094 | 1.135 | 0.866^***^ | 0.851 | 0.881 | 1.088^***^ | 1.067 | 1.108 |
| Other races/  ethnicities | 1.655^***^ | 1.609 | 1.702 | 1.418^***^ | 1.374 | 1.464 | 1.280^***^ | 1.242 | 1.32 | 1.407^***^ | 1.362 | 1.453 |

***Note:*** *This table presents the results from four sets of logistic regression analysis combined with linear splines without weighting variable. Model 1: unadjusted model. Model 2: adjusted for demographic and socioeconomic variables Insurance type, gender, age at admission, median household income for patient's ZIP Code, location. Model 3: adjusted for clinical variables. Model 4: fully adjusted model for all demographic, socioeconomic and clinical variables. * p<0.05, ** p<0.01, *** p<0.001. CI – Confidence intervals.*

Full results (Using Model 4 from Appendix 2):

| pri_bariatric | Odds Ratio | Std. Err. | z | P>z | 95% Confidence  Interval | |
| --- | --- | --- | --- | --- | --- | --- |
|  |  |  |  |  |  |  |
| 1.native | 0.692 | 0.028 | -9.240 | <0.001 | 0.640 | 0.748 |
| 1.black | 0.738 | 0.006 | -37.350 | <0.001 | 0.727 | 0.750 |
| 1.asian | 0.722 | 0.024 | -9.840 | <0.001 | 0.677 | 0.771 |
| 1.hispanic | 1.088 | 0.010 | 8.770 | <0.001 | 1.067 | 1.108 |
| 1.other_ethnic | 1.407 | 0.023 | 20.580 | <0.001 | 1.362 | 1.453 |
| 1.medicaid | 0.779 | 0.010 | -19.040 | <0.001 | 0.759 | 0.799 |
| 1.medicare | 1.122 | 0.015 | 8.840 | <0.001 | 1.094 | 1.151 |
| 1.private_ins | 2.446 | 0.028 | 78.460 | <0.001 | 2.392 | 2.501 |
|  |  |  |  |  |  |  |
| FEMALE#agecat10 |  |  |  |  |  |  |
| Male#40-46y | 0.949 | 0.018 | -2.770 | 0.006 | 0.915 | 0.985 |
| Male#47-51y | 0.887 | 0.017 | -6.130 | <0.001 | 0.853 | 0.921 |
| Male#52-55y | 0.966 | 0.020 | -1.670 | 0.095 | 0.927 | 1.006 |
| Male#56-58y | 0.879 | 0.019 | -5.870 | <0.001 | 0.842 | 0.918 |
| Male#59-62y | 0.963 | 0.022 | -1.630 | 0.103 | 0.921 | 1.008 |
| Male#63-65y | 1.057 | 0.029 | 2.010 | 0.045 | 1.001 | 1.115 |
| Male#66-69y | 1.119 | 0.031 | 4.060 | <0.001 | 1.060 | 1.181 |
| Male#70-75y | 1.019 | 0.039 | 0.490 | 0.625 | 0.946 | 1.097 |
| Male#>=76y | 0.260 | 0.027 | -13.150 | <0.001 | 0.212 | 0.317 |
| Female#<= 39y | 1.996 | 0.030 | 45.810 | <0.001 | 1.938 | 2.056 |
| Female#40-46y | 1.765 | 0.027 | 36.920 | <0.001 | 1.712 | 1.819 |
| Female#47-51y | 1.665 | 0.026 | 32.470 | <0.001 | 1.614 | 1.717 |
| Female#52-55y | 1.932 | 0.031 | 40.620 | <0.001 | 1.872 | 1.994 |
| Female#56-58y | 1.865 | 0.031 | 37.580 | <0.001 | 1.805 | 1.926 |
| Female#59-62y | 1.801 | 0.032 | 33.510 | <0.001 | 1.740 | 1.864 |
| Female#63-65y | 1.917 | 0.038 | 32.700 | <0.001 | 1.844 | 1.993 |
| Female#66-69y | 1.588 | 0.033 | 22.010 | <0.001 | 1.524 | 1.655 |
| Female#70-75y | 1.111 | 0.032 | 3.700 | <0.001 | 1.051 | 1.175 |
| Female#>=76y | 0.180 | 0.014 | -21.700 | <0.001 | 0.155 | 0.211 |
|  |  |  |  |  |  |  |
| income1 | 0.571 | 0.005 | -60.480 | <0.001 | 0.561 | 0.581 |
| income2 | 0.708 | 0.006 | -39.350 | <0.001 | 0.696 | 0.721 |
| income3 | 0.789 | 0.007 | -28.420 | <0.001 | 0.776 | 0.802 |
|  |  |  |  |  |  |  |
| location |  |  |  |  |  |  |
| Large metro | 1.000 | 0.008 | -0.040 | 0.971 | 0.984 | 1.015 |
| Medium metro | 1.159 | 0.010 | 17.870 | <0.001 | 1.140 | 1.178 |
| Small metro | 1.041 | 0.012 | 3.650 | <0.001 | 1.019 | 1.064 |
| Micropolitan | 1.666 | 0.021 | 40.300 | <0.001 | 1.625 | 1.708 |
| Not metropolitan or Micropolitan | 1.428 | 0.020 | 25.090 | <0.001 | 1.389 | 1.468 |
|  |  |  |  |  |  |  |
| ACCI | 0.575 | 0.002 | -191.800 | <0.001 | 0.572 | 0.578 |
| 1.diab_type2 | 1.656 | 0.012 | 70.270 | <0.001 | 1.633 | 1.680 |
| 1.hypertension | 1.184 | 0.007 | 27.320 | <0.001 | 1.170 | 1.199 |
| 1.private_hosp | 1.228 | 0.013 | 20.130 | <0.001 | 1.204 | 1.253 |
| 1.urban_hosp | 2.854 | 0.050 | 59.780 | <0.001 | 2.757 | 2.953 |
| 1.teaching_hosp | 1.306 | 0.008 | 43.210 | <0.001 | 1.290 | 1.322 |
|  |  |  |  |  |  |  |
| HOSP_BEDSIZE |  |  |  |  |  |  |
| Medium | 1.107 | 0.009 | 11.880 | <0.001 | 1.089 | 1.126 |
| Large | 0.933 | 0.008 | -8.570 | <0.001 | 0.919 | 0.948 |
|  |  |  |  |  |  |  |
| YEAR | 1.023 | 0.001 | 18.090 | <0.001 | 1.021 | 1.026 |
| _cons | 0.000 | 0.000 | -19.450 | <0.001 | 0.000 | 0.000 |

**Appendix 3**

**Appendix 4**

**Table 4. Main logistic model on bariatric surgery, fully adjusted (Model 4)**

| pri_bariatric | Odds Ratio | Std. Err. | z | P>z | 95% Confidence  Interval | |
| --- | --- | --- | --- | --- | --- | --- |
|  |  |  |  |  |  |  |
| 1.native | 0.722 | 0.033 | -7.170 | <0.001 | 0.661 | 0.790 |
| 1.black | 0.711 | 0.007 | -36.300 | <0.001 | 0.699 | 0.725 |
| 1.asian | 0.838 | 0.031 | -4.750 | <0.001 | 0.779 | 0.902 |
| 1.hispanic | 1.077 | 0.012 | 6.470 | <0.001 | 1.053 | 1.102 |
| 1.other_ethnic | 1.365 | 0.027 | 15.900 | <0.001 | 1.314 | 1.418 |
| REF white |  |  |  |  |  |  |
| 1.medicaid | 0.814 | 0.013 | -13.090 | <0.001 | 0.790 | 0.840 |
| 1.medicare | 1.252 | 0.019 | 14.930 | <0.001 | 1.215 | 1.289 |
| 1.private_ins | 2.671 | 0.036 | 72.900 | <0.001 | 2.601 | 2.743 |
| REF others |  |  |  |  |  |  |
|  |  |  |  |  |  |  |
| FEMALE#agecat10 |  |  |  |  |  |  |
| 0 2 | 0.921 | 0.018 | -4.180 | <0.001 | 0.886 | 0.957 |
| 0 3 | 0.817 | 0.017 | -9.630 | <0.001 | 0.784 | 0.851 |
| 0 4 | 0.866 | 0.020 | -6.300 | <0.001 | 0.829 | 0.906 |
| 0 5 | 0.739 | 0.019 | -11.910 | <0.001 | 0.703 | 0.777 |
| 0 6 | 0.921 | 0.023 | -3.250 | 0.001 | 0.876 | 0.968 |
| 0 7 | 0.977 | 0.031 | -0.730 | 0.464 | 0.918 | 1.040 |
| 0 8 | 1.107 | 0.033 | 3.410 | 0.001 | 1.044 | 1.174 |
| 0 9 | 0.902 | 0.039 | -2.410 | 0.016 | 0.829 | 0.981 |
| 0 10 | 0.161 | 0.021 | -14.120 | <0.001 | 0.125 | 0.208 |
| 1 1 | 2.321 | 0.037 | 52.710 | <0.001 | 2.250 | 2.395 |
| 1 2 | 1.921 | 0.031 | 39.800 | <0.001 | 1.860 | 1.983 |
| 1 3 | 1.666 | 0.028 | 29.970 | <0.001 | 1.611 | 1.723 |
| 1 4 | 1.837 | 0.033 | 33.880 | <0.001 | 1.773 | 1.903 |
| 1 5 | 1.505 | 0.029 | 21.220 | <0.001 | 1.449 | 1.563 |
| 1 6 | 1.595 | 0.032 | 23.330 | <0.001 | 1.534 | 1.659 |
| 1 7 | 1.517 | 0.036 | 17.380 | <0.001 | 1.447 | 1.589 |
| 1 8 | 1.428 | 0.034 | 14.950 | <0.001 | 1.363 | 1.496 |
| 1 9 | 0.808 | 0.028 | -6.090 | <0.001 | 0.754 | 0.865 |
| 1 10 | 0.103 | 0.011 | -21.740 | <0.001 | 0.084 | 0.127 |
|  |  |  |  |  |  |  |
| income1 | 0.543 | 0.006 | -56.860 | <0.001 | 0.531 | 0.554 |
| income2 | 0.696 | 0.007 | -35.730 | <0.001 | 0.683 | 0.710 |
| income3 | 0.784 | 0.008 | -25.100 | <0.001 | 0.769 | 0.799 |
| REF income quartile 4 |  |  |  |  |  |  |
|  |  |  |  |  |  |  |
| location |  |  |  |  |  |  |
| Fringe counties of metro areas of >=1 million population (large metro) | 0.975 | 0.009 | -2.740 | 0.006 | 0.957 | 0.993 |
| Counties in metro areas of 250,000-999,999 population (medium metro) | 1.164 | 0.011 | 15.950 | <0.001 | 1.143 | 1.186 |
| Counties in metro areas of 50,000-249,999 population (small metro) | 1.047 | 0.013 | 3.590 | <0.001 | 1.021 | 1.073 |
| Micropolitan counties | 1.716 | 0.025 | 37.530 | <0.001 | 1.668 | 1.765 |
| Not metropolitan or micropolitan counties | 1.451 | 0.023 | 23.080 | <0.001 | 1.406 | 1.498 |
| REF central counties |  |  |  |  |  |  |
|  |  |  |  |  |  |  |
| ACCI | 0.593 | 0.002 | -159.430 | <0.001 | 0.589 | 0.597 |
| 1.diab_type2 | 1.702 | 0.014 | 66.860 | <0.001 | 1.676 | 1.729 |
| 1.hypertension | 1.348 | 0.014 | 29.140 | <0.001 | 1.321 | 1.376 |
| 1.private_hosp | 1.196 | 0.014 | 15.360 | <0.001 | 1.169 | 1.223 |
| 1.urban_hosp | 2.950 | 0.058 | 54.610 | <0.001 | 2.838 | 3.067 |
| 1.teaching_hosp | 1.306 | 0.009 | 37.810 | <0.001 | 1.288 | 1.324 |
|  |  |  |  |  |  |  |
| HOSP_BEDSIZE |  |  |  |  |  |  |
| Medium | 1.146 | 0.011 | 13.600 | <0.001 | 1.123 | 1.168 |
| Large | 0.970 | 0.009 | -3.290 | 0.001 | 0.952 | 0.988 |
| REF small |  |  |  |  |  |  |
|  |  |  |  |  |  |  |
| YEAR | 1.020 | 0.001 | 14.420 | <0.001 | 1.018 | 1.023 |
| _cons | 0.000 | 0.000 | -15.750 | <0.001 | 0.000 | 0.000 |
